# Breast Metastasis From a Non-small Cell Lung Cancer: A Case Report

**DOI:** 10.7759/cureus.7460

**Published:** 2020-03-29

**Authors:** Mohammed Amine Guerrouaz, Soumiya Samba, Ahmed BenSghier, Ali Sbai, Loubna Mezouar

**Affiliations:** 1 Department of Radiation Therapy, Centre Hospitalier Universitaire Mohammed Vi, Oujda, MAR; 2 Department of Radiation Oncology, Centre Hospitalier Universitaire Mohammed Vi, Oujda, MAR

**Keywords:** breast, metastasis, lung cancer, case report

## Abstract

Although primary breast cancer (BC) is the most frequent cancer in the world, their mammary metastases are rare. In this paper, we report the case of a 51-year-old male patient diagnosed with a mammary lump and an intracranial hypertension syndrome. Mammography showed the presence of an irregular opacity without microcalcifications. After a cytological and immunohistochemical examination, the breast biopsy showed an adenocarcinoma of pulmonary origin. Computed tomography (CT) scans of the cerebrum, cervicothoracic spine, and abdomen showed a right apical pulmonary tumor associated with Barety's lymphadenopathy, as well as the presence of a mass in the right breast and secondary brain lesions. The patient received total brain irradiation which resulted in a marked clinical improvement. Afterward, he was treated with six cycles of palliative chemotherapy based on carboplatin area under the curve (AUC) 6 and paclitaxel, 175 mg/m² every 21 days, with a good response. After two months, the patient presented with a progression and he was treated with second-line chemotherapy. However, after three chemotherapy courses, the disease rapidly progressed and the patient was altered which required his admission to the palliative care department. The patient died 40 days later. This case shows the poor prognostic survival outcomes in this population of patients.

## Introduction

Secondary breast tumors are rare. Breast metastases account for 0.4% to 2% of all mammary cancers. Melanomas, lymphomas, and lung cancers are most often the primary tumors [1]. To date, more than 400 cases of mammary metastases have been reported in the literature [2-3]. In this case report, we illustrate a new case of a patient with lung carcinoma with breast metastasis and report the clinicopathological features and therapeutics interventions, as well as the outcomes of this aggressive disease.

## Case presentation

**Figure 1:**
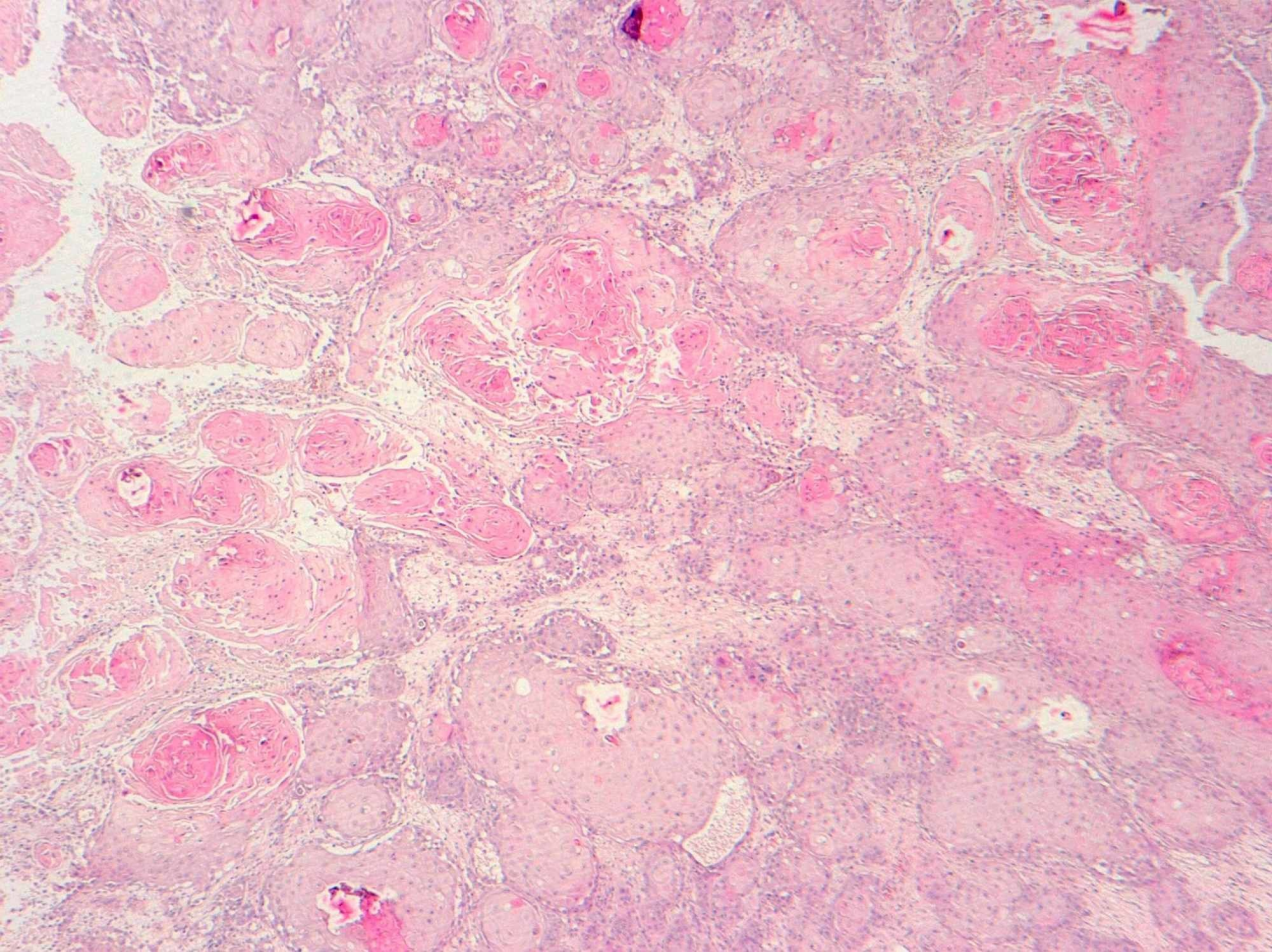
Microphotography showing a carcinomatous proliferation made of nests, often centred by keratin pearls (hematoxylin & eosin, 40X)

A 51-year-old male patient was admitted to our radiation oncology unit with headache, vomiting, and right hemiplegia as part of an intracranial hypertension syndrome with no previous medical history. The initial physical examination found a mammary mass of 5 cm in the right breast with no ipsilateral axillary lymph node enlargement. A mammogram showed an irregular opacity and hypoechogenic mass on ultrasound without microcalcifications measuring 45 mm and without axillary lymph node abnormality (classified as Breast Imaging-Reporting and Data System (BI-RADS) 4). A biopsy of the mammary mass was performed, and the histopathological findings showed an unspecified tumor (Figure 1).

**Figure 2:**
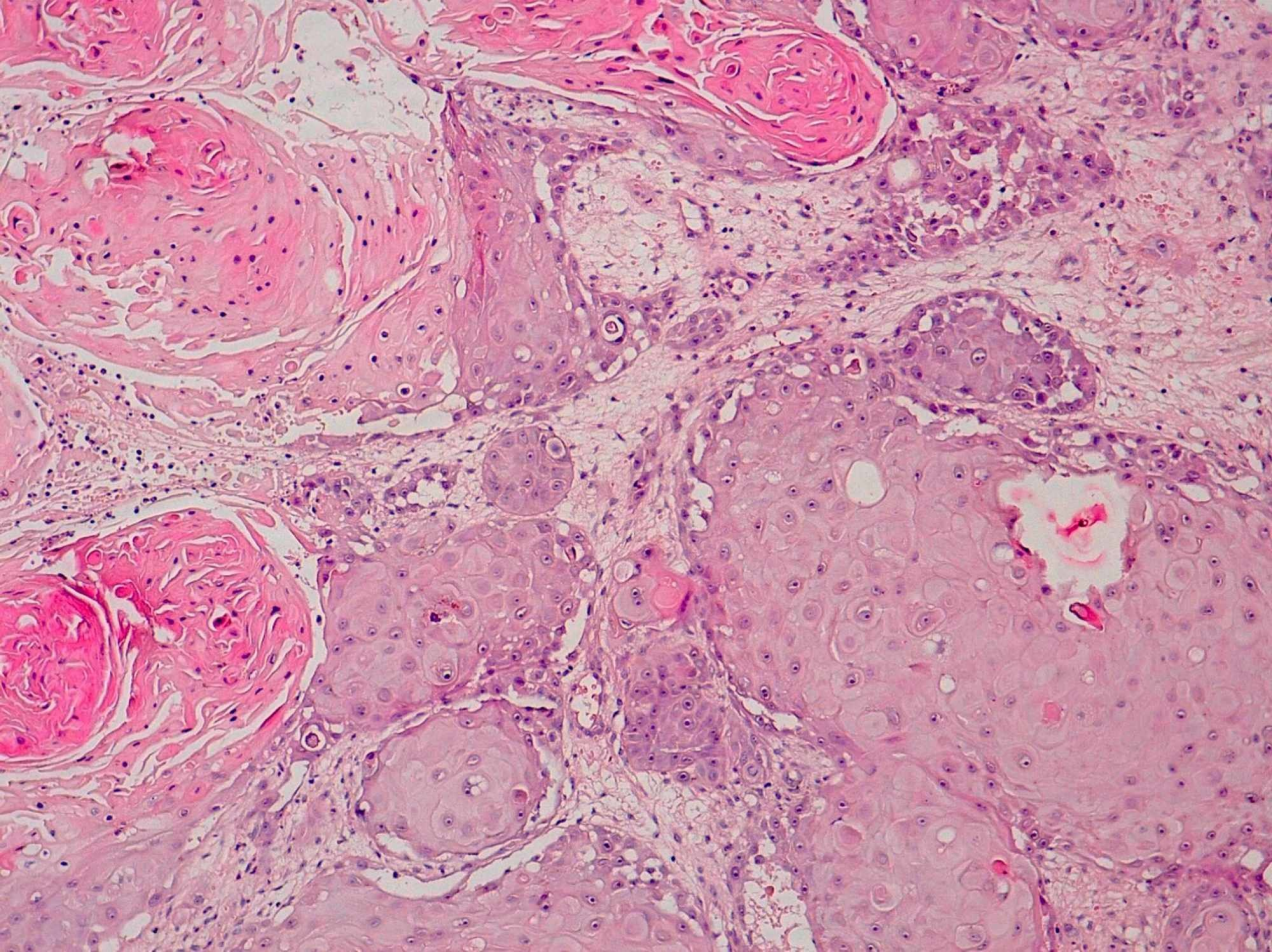
Microphotography at higher magnification showing carcinomatous nests made of eosinophilic large polygonal cells The nuclei have prominent nucleoli (hematoxylin & eosin, 200X)

After a higher magnification, it showed carcinomatous features which were mainly characterized by large eosinophilic polygonal cells with several nucleoli (Figure 2).

**Figure 3:**
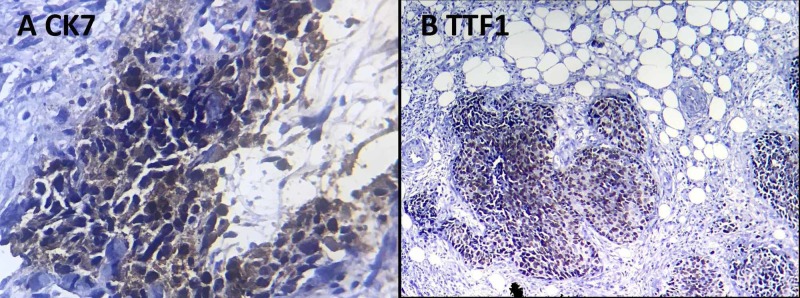
The tumor cells express CK 7 (A) and TTF-1 (B). CK 7: cytokeratin 7; TTF1: thyroid transcription factor 1

**Figure 4:**
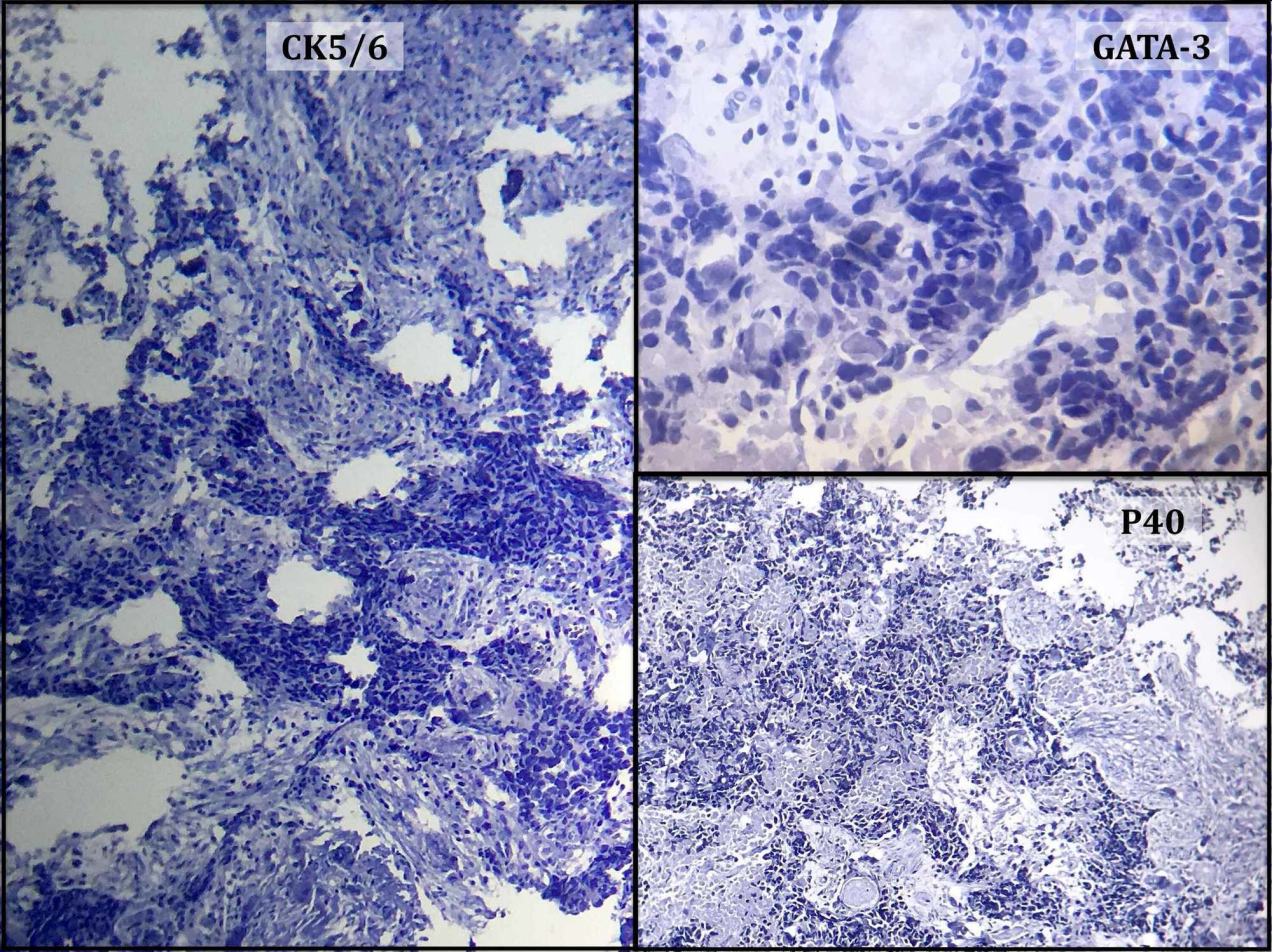
The tumor cells do not express GATA binding protein 3 (GATA3), cytokeratin (CK) 5/6 or P40 antigens

Immunohistochemistry was performed and showed an adenocarcinoma with positive thyroid transcription factor 1 (TTF1) which is consistent with a lung or thyroid cancer origin (Figures 3-4).

Cervicothoracic and abdominal computed tomography (CT) scans showed a right apical lung tumor associated with Barety's space lymph node. It also revealed a right breast mass, which was already found on the mammogram, and bilateral adrenal nodes in favor of secondary lesions. Moreover, a cerebral CT scan objectified several bilateral temporoparietal lesions with a right cerebellar location. This was classified as Stage IV disease.

**Figure 5:**
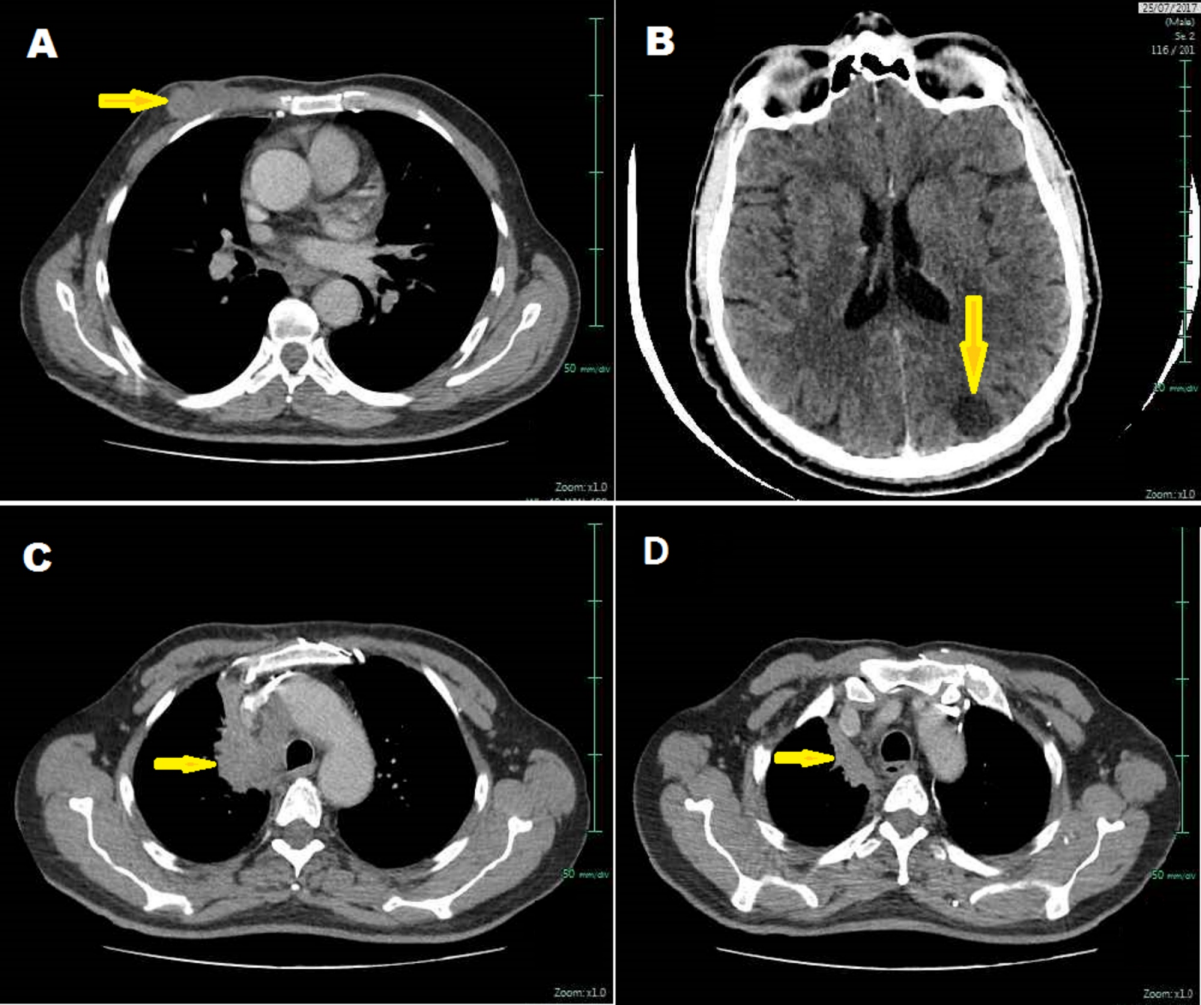
Cerebral, cervicothoracic, and abdominal computed tomography (CT) scans A) right breast mass, B) brain metastasis, C-D) right apical lung tumor

The patient received total brain irradiation at a dose of 30 Gray in 10 fractions of 3 Gray per fraction over two weeks with remarkably improved clinical outcomes (headache, vomiting, and right hemiplegia). Later, he was treated with palliative chemotherapy based on carboplatin (AUC 6) and paclitaxel (175 mg/m²) per cycle of 21 days. After the third cycle, a CT scan evaluation showed an incomplete radiological remission estimated at more than 70% (Figure 5C-D). The evaluation after the sixth cycle of chemotherapy showed stable disease. After medical assessment two months later, a CT scan detected local breast and cerebral progression (Figure 5 A-B). He was, therefore, treated with second-line chemotherapy with gemcitabine monotherapy, 1,000 mg/m² on Days 1 and 8 every 21 days (delivered on Days 1 and 8 and wait 14 days to repeat the same cure). During follow-up, the patient's general condition deteriorated rapidly (World Health Organization (WHO) score of 3). He was referred for palliative care and died six weeks later.

## Discussion

BC is the most common cancer worldwide. However, breast metastases from extra-mammary cancers are extremely rare, representing less than 2% of all malignant breast tumors [2-6]. Breast metastases may be the first symptom revealing a primary lung tumor in 8% to 33% of reported cases [7]. These metastases may be related to ipsilateral axillary lymph nodes in more than 50% of cases. The most common type of primary cancer is melanoma [5]. Up to now, the study with the largest enrollment was conducted by Georgiannos et al. which included 60 cases of breast metastases (0.43%), 95% of whom were women with an age ranging from 12 to 90 years [2]. In addition, the main primary tumors were cutaneous melanomas, and as in our case, large and small cell lung cancer, which is the second cause of these intramammary metastases. However, there were other reported primary tumors, such as endometrial/pancreatic adenocarcinomas and retinal melanoma. Other locations for these metastases include uterine, gastric, and rectal cancers and non-Hodgkin's lymphoma [6, 8]. For the latter, it includes 17% of all breast metastases [8].

Regarding mammary metastases from primary gastric cancer, they have been identified by Krichen et al. in the case of metastatic gastric adenocarcinoma of the ovary; the primary tumor and ovarian metastases were surgically removed [9]. A metastatic relapse was detected as an isolated mammary metastasis of the same histological type as gastric cancer after four months. It was subsequently removed, but the disease was marked by the development of cutaneous lymphangitis of the chest wall and the occurrence of lymph nodes and bone metastases. This is not the only case of gastric cancer. Boutis et al. also reported a series of 25 cases of gastric cancer associated with breast metastases of which 13/25 had a form of signet-ring cell carcinoma [10]. Moreover, tumors that can metastasize to the breast also include neuroendocrine tumors. A case series (n = 24) of secondary neuroendocrine tumors of the breast reported by Upalakalin et al. showed that breast metastasis was the primary tumor site in nine cases [11].

The clinical presentation of these tumors most often is a well-rounded, hard, and painless lump located at the upper outer quadrant [6, 9, 12-14]. This lump may be associated with axillary metastases and other metastatic lesions [14-16].

Mammography found regular or slightly irregular round nodules containing no microcalcifications, except in the case of metastases of ovarian origin [13-16]. On breast ultrasound, they appear hypoechoic without acoustic shadowing. On histopathological examination, intramammary metastases have different characteristics as compared to primary BC. In fact, most intramammary metastases have similar histology of the tumor from which they originate, such as in the case of metastases from ovarian cancer that have intra-metastatic microcalcifications [4, 14, 16]. However, one can be mistaken in making the diagnosis of primitive breast cancer [17-18]. To overcome this problem, it is recommended to use immunohistochemistry to accurately differentiate primary cancers from metastases. Usually, for primitive breast tumors, epithelial biomarkers, such as CK-7 and CK-20, are positive and negative, respectively, and hormone receptors are positive. In the case of metastasis, CK-7 and hormone receptors are negative [14-15]. Additional tumor markers, such as TTF1 (lung cancer or thyroid cancer as in our case) and desmin (soft tissue tumors), can be used to guide the diagnosis of the primary tumor [19]. On the other hand, the elevation of some serum markers can be useful to make the diagnosis, including carcinoembryonic antigen (CEA) (colon cancer), CA-19-9 (pancreatic cancer), and CA-125 (ovarian or gastrointestinal cancer).

The management of mammary metastases must be part of the treatment of primary cancer and is based on palliative chemotherapy which can be combined with palliative radiotherapy in the case of emergency, such as metastatic spinal cord compression [4]. Surgical treatment is not indicated in these cases, except for diagnostic purposes or, more rarely, in the case of a locally advanced mammary metastasis [15]. To date, there are no studies that support axillary dissection in cases of mammary metastasis associated with positive axillary lymph nodes which may significantly increase postoperative morbidity [20]. The prognosis of this entity is generally poor and depends on the primary cancer. The median overall survival of these patients with breast metastases is poor [15]. Therefore, additional studies on emerging treatments to improve outcomes in this rare presentation are still awaited.

## Conclusions

Breast metastases are rare tumors. They are difficult to diagnose that's why it is recommended to use immunohistochemistry to accurately differentiate primary cancers from metastases. They require multidisciplinary assessment for better management. Their treatments are mainly based on palliative chemotherapy and surgical resections should be avoided. Their prognostic evolutions depend on the primary tumor and are generally poor.
